# Generation of microdissected DNA probes from metaphase chromosomes when chromosome identification by routine staining is impossible

**DOI:** 10.18699/VJ20.46-o

**Published:** 2020-08

**Authors:** K.S. Zadesenets, N.B. Rubtsov

**Affiliations:** Institute of Cytology and Genetics of Siberian Branch of the Russian Academy of Sciences, Novosibirsk, Russia; Institute of Cytology and Genetics of Siberian Branch of the Russian Academy of Sciences, Novosibirsk, Russia

**Keywords:** metaphase chromosome microdissection, Whole Chromosome Paints, FISH, sequence-independent polymerase chain reaction, микродиссекция метафазных хромосом, микродиссекционные ДНК-пробы, флуоресцентная in situ гибридизация, сиквенс-независимая полимеразная цепная реакция

## Abstract

Application of microdissected DNA libraries and DNA probes in numerous and various modern molecular cytogenetic studies showed them as an efficient and reliable tool in the analysis of chromosome reorganization during karyotypic evolution and in the diagnosis of human chromosome pathology. An important advantage of DNA probe generation by metaphase chromosome microdissection followed by sequence-independent polymerase chain reaction in comparison with the method of DNA probe generation using chromosome sorting is the possibility of DNA probe preparation from chromosomes of an individual sample without cell line establishment for the production of a large number of metaphase chromosomes. One of the main requirements for successful application of this technique is a possibility for identification of the chromosome of interest during its dissection and collection of its material from metaphase plates spread on the coverslip. In the present study, we developed and applied a technique for generation of microdissected DNA probes in the case when chromosome identification during microdissection appeared to be impossible. The technique was used for generation of two sets of Whole Chromosome Paints (WCPs) from all chromosomes of two species of free-living flatworms in the genus Macrostomum, M. mirumnovem and M. cliftonensis. The single-copy chromosome technique including separate collection of all chromosomes from one metaphase plate allowed us to generate WCPs that painted specifically the original chromosome by Chromosome In Situ Suppression Hybridization (CISS-Hybridization). CISS-Hybridization allowed identifying the original chromosome(s) used for DNA probe generation. Pooled WCPs derived from homologous chromosomes increased the intensity and specificity of chromosome painting provided by CISS-Hybridization. In the result, the obtained DNA probes appeared to be good enough for application in our studies devoted to analysis of karyotypic evolution in the genus Macrostomum and for analysis of chromosome rearrangements among the worms of laboratory cultures of M. mirumnovem.

## Abstract (ru)

При проведении многочисленных и разнообразных молекулярно-цитогенетических исследо- ваний микродиссекционные ДНК-библиотеки и ДНК-пробы зарекомендовали себя как надежный и эффек- тивный инструмент как в диагностике и анализе хромосомных патологий человека, так и в работах, посвя- щенных изучению реорганизации хромосом в ходе кариотипической эволюции. Важным преимуществом микродиссекционных ДНК-проб перед хромосомоспецифичными ДНК-пробами, полученными с помощью хромосомного сортинга, является возможность их приготовления из хромосомного материала индивидуальных животных без дополнительного этапа создания клеточных культур, предназначенных для произ- водства большого числа метафазных хромосом. Одно из основных условий успешного использования микродиссекционной техники – идентификация целевой хромосомы на препаратах метафазных хромосом, что позволяет, используя микроманипуляционную технику, осуществлять сбор непосредственно ее материала с цитологических препаратов. В настоящей работе предложена технология создания ДНК-проб для индивидуальных хромосом даже в том случае, когда рутинное окрашивание не дает провести их надежную иден- тификацию. Представленный подход апробирован при получении наборов хромосомоспецифичных ДНК- проб для хромосом двух видов свободноживущих плоских червей рода Macrostomum – M. mirumnovem и M. cliftonensis. Кариотипы этих видов содержат три пары мелких, близких по размеру метацентрических хро- мосом, надежная идентификация которых после окрашивания красителем Гимза оказалась невозможной. Раздельный сбор всех метафазных хромосом из одной метафазной пластинки с последующей амплифика- цией их ДНК позволил создать ДНК-пробы, специфически окрашивающие исходные хромосомы при прове- дении даже частичной супрессионной гибридизации in situ. При анализе результатов такой супрессионной гибридизации in situ идентифицированы хромосомы, из которых были получены ДНК-пробы. Последующее пулирование ДНК-проб, созданных из гомологичных хромосом, способствовало увеличению интенсивно- сти их специфического окрашивания при проведении их супрессионной гибридизации in situ. Это, в свою очередь, обеспечило возможность успешного применения предлагаемого подхода в экспериментах, по- священных изучению кариотипической эволюции в роде Macrostomum, а также при анализе хромосомных перестроек, имеющих место в лабораторных культурах M. mirumnovem.

## Introduction

Comparative cytogenetics as a special area in the modern biology arose after the development of methods for high-quality metaphase chromosome preparation. Its progress is associated mostly with the development of techniques for chromosomes and chromosome regions identification. Since the 1970s, researchers successfully used the GTG-banding method for comparative cytogenetic analysis of chromosomes of different species of mammals and birds (Graphodatsky et al., 2000). The next step in development of comparative cytogenetics was the homeologous gene assignment to chromosomes or chromosome regions in different species of mammals that served as markers of their homeology. In the first studies devoted to gene assignments to the chromosomes, the panels of interspecific hybrids of somatic cells were used (Rubtsov et al., 1981). The obtained data were combined with a comparison of GTG-banding patterns of chromosomes containing homeologous genes (Rubtsov et al., 1988).

Significant progress in comparative cytogenetics has been associated with the development of fluorescent in situ hybridization (FISH) technique for nucleic acids in the early 1980s (Bauman et al., 1980). This technique made it possible to localize cloned DNA fragments precisely to small chromosomal regions, and then specifically paint the whole chromosomes or extended chromosome regions (Nesterova et al., 1991). One of the pivotal moments in the development of comparative cytogenetics appeared to be the development of physical isolation of chromosomal material. Two different techniques, namely metaphase chromosome microdissection and chromosome flow sorting, are used for generation of the whole chromosome or partial chromosome paints (WCPs and PCPs, respectively). These paints are generated from isolated chromosomal material through sequence-independent DNA amplification in a polymerase chain reaction with partially degenerated MW6 primer, or by using the special WGA-kits (whole genome amplification). Chromosome in situ suppression hybridization (CISS-hybridization) painted specifically the original chromosome or chromosome region and also homeologous chromosomes and correspondent region in related species (Ferguson-Smith, Trifonov, 2007). The quality of such WCPs depends on the efficiency of DNA amplification of the collected chromosomal material, the number of isolated chromosome copies used on the start of DNA amplification, and the quality of DNA of the collected chromosomal material. The high quality of whole chromosome paints can be achieved by collecting many hundreds of chromosome copies using flow sorting. In the case of microdissection, the number of obtained chromosome copies is limited due to the high complexity of the microdissection procedure. The problems of identification chromosome of interest could make the application of chromosome microdissection technology even more complicated task.

In the molecular cytogenetic analysis of chromosomes of free-living worms of the genus Macrostomum, we encountered had to solve the problem of whole chromosome paints generation from chromosomes that avoid reliable identification after chromosome staining. The karyotypes of species belonging to the genus Macrostomum can be divided into three groups based on their chromosome number and morphology. The karyotypes of species from two groups (2n = 6 and 2n = 12) consist of small metacentric chromosomes suggesting that a recent whole genome duplication (WGD) could take place in the evolution providing species with chromosome number 2n = 12. This hypothesis is in a good agreement with the results of molecular cytogenetic analysis of asymmetric karyotypes of M. lignano and M. janickei species. In the karyotypes of these species, there are clear traces of a recent WGD event (Zadesenets et al., 2017a, b). In addition to a WGD in their evolution, there was a fusion of one haploid set of ancestral chromosomes into one large metacentric chromosome (Zadesenets et al., 2017a, b).

The hypothesis of chromosome number doubling in a result of WGD can be verified by generation of WCPs from individual chromosomes of the Macrostomum species having the 2n = 12 karyotype and further CISS-hybridization on metaphase chromosomes of the species. The specific painting of two pairs of paralogous chromosomes with the WCP derived from individual chromosome would indicate to a recent duplication of this one in karyotype evolution of studied species. The same results obtained for all chromosomes will confirm the hypothesis of the WGD that recently took place in the genome evolution of analyzed species. The high level of similarity of all chromosomes have complicated the generation of specific WCPs that could be applied for such a study. Such similarity of the morphology and size of all chromosomes was revealed in all currently karyotyped species of the genus Macrostomum with the chromosome set 2n = 12 (Zadesenets et al., 2020).

We investigated the karyotypes of new Macrostomum species that potentially could be involved in these studies. Additionally, we developed the method for the generation of WCPs that painted original chromosomes in species with morphologically indistinguishable chromosomes.

## Material and methods

**Laboratory cultures of the free-living Macrostomum worms.** Laboratory cultures of M. cliftonensis and M. mirumnovem were kindly provided by Dr. Lukas Schärer (Zoological Institute, University of Basel, Switzerland). The outbred cultures of M. cliftonensis and M. mirumnovem were maintained in the laboratory of Institute of Cytology and Genetics of Siberian Branch of the Russian Academy of Sciences. The karyotype of M. cliftonensis (2n = 6) consists of three pairs of small metacentric chromosomes of similar size and morphology (Zadesenets et al., 2020). Karyotyping of M. mirumnovem revealed a high karyotypic diversity, with the most common chromosome number 2n = 9 (Zadesenets et al., 2020). In this study, we used only the worms with the 2n = 9 karyotype.

**Metaphase chromosome slide preparation.** Chromosome slide preparation was carried out according to the previously published protocol for single-worm karyotyping (Zadesenets et al., 2016). To describe the karyotype, we analyzed at least ten metaphase plates per each specimen. For microdissection, chromosome slides were prepared from chromosome suspension, as described earlier (Zadesenets et al., 2016).

**Metaphase chromosome staining.** For routine karyotyping, chromosomes were stained with DAPI (4’, 6-diamidino- 2- phenylindole) (Vector Laboratories, USA) under the standard protocol. For microdissection, chromosomes were stained with 0.1 % Giemsa solution for 3 min at room temperature (RT). After staining, they were rinsed in distilled water and air-dried. After drying, the chromosomes should remain soft enough for effectively cutting with an extended glass needle.

**Microscopy.** Microimages of metaphase chromosomes after DAPI-staining and FISH were captured using a CCD-camera installed on an Axioplan 2 Plus microscope (ZEISS, Germany) equipped with a fluorescence filter cube set, #49, #10 and #15 (ZEISS, Germany). AxioVision (ZEISS, Germany) or ISIS4 (METASystems GmbH, Germany) software was applied for caption and analysis of chromosome microimages. Microscopy was performed at the Center for Microscopic Analysis of Biological Objects of SB RAS (Novosibirsk, Russia).

**Metaphase chromosome microdissection and whole chromosome paint generation.** In general, metaphase chromosome microdissection in species of the genus Macrostomum and DNA sequence-independent amplification of DNA of dissected material for WCP generation were mainly described previously (Zadesenets et al., 2016, 2017a, b). Briefly, only complete metaphase plates were used in microdissection experiments. The material in the metaphase plate had to be well spread without chromosome contacts and overlapping. Such quality of chromosome spreading allowed us to carefully isolate all chromosomes from the metaphase plate and transfer the material of each chromosome into a separate tube. For microdissection of the M. mirumnovem chromosomes, we used only individuals with the 2n = 9 karyotype. Their chromosome set included the unpaired largest metaphase chromosome MMI1, the pair of large metacentrics MMI2, and three pairs of small metacentric chromosomes MMI3–MMI5. Under microscopic control, the material of the isolated chromosome was transferred to 40 nl of the reaction mixture (Zadesenets et al., 2016), placed in the extended siliconized tip of the Pasteur pipette. Microscopic control guaranteed reliable and complete transfer of isolated chromosome. Then, its material was treated with proteinase K and transferred to 10 μl of the reaction mixture within a 0.5 ml Eppendorf Safe-Lock microcentrifuge tube. Further, DNA preparation for amplification and the amplification itself was performed according to the previously described protocol (Zadesenets et al., 2016, 2017a, b). After polymerase chain reaction (PCR), the resulting DNA product was labeled in 20 additional PCR cycles in the presence of Flu-12-dUTP [fluorescein-5(6)-carboxamidocaproyl-[5(3-aminoallyl)2′- deoxyuridine-5′-Triphosphate] (Biosan, Novosibirsk, Russia) or TAMRA-5-dUTP (5-tetramethylrhodamine-dUTP) (Biosan) using the Whole Genome Amplification 3 Kit (WGA3, Sigma-Aldrich, USA) (Zadesenets et al., 2016, 2017a, b). The WCPs were tested by CISS-hybridization with metaphase chromosomes of the original species.

**CISS-hybridization with metaphase chromosomes of M. cliftonensis and M. mirumnovem.** Due to the small body size of M. cliftonensis and M. mirumnovem (the mean body length of adult worms does not exceed 1.22 and 1.17 mm, respectively) (Schärer et al., 2020), it was impossible to obtain a sufficient amount of Cot1/Cot2 DNA (fraction of highly repetitive DNA) for routine CISS-hybridization. Previously we developed a modification of the CISS-hybridization with WCPs generated from microdissected chromosomes of some Macrostomum species (Zadesenets et al., 2017a, b, 2020). This CISS-hybridization gave different painting patterns in different chromosomes regions, depending on their enrichments with DNA repeats. The euchromatic regions at the original chromosome showed specific painting patterns. Less intense fluorescence was observed at other chromosome euchromatic regions containing dispersed DNA repeats. In contrast, more intense signals were found in the heterochromatic regions enriched for DNA repeats homologous to those in the WCPs.

## Results and discussion

**Fig. 1.**
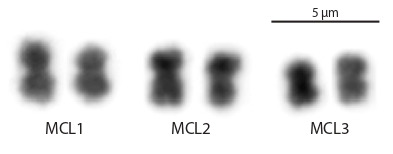
The karyotype of M. cliftonensis (2n = 6) consists of three pairs of small metacentric chromosomes showing similar size and morphology.

**Chromosomes of M. cliftonensis.** Before microdissection, we repeatedly checked the karyotypes of randomly chosen 100 specimens of M. cliftonensis. All metaphase plates in analyzed samples contained the standard for M. cliftonensis chromosome set, 2n = 6, consisting of three pairs of small metacentric chromosomes (Fig. 1).

**Generation and testing of WCPs derived from metaphase chromosomes of M. cliftonensis.** For obtaining of metaphase plates of M. cliftonensis, suspension of mitotic cells was dropped on a cold, wet glass coverslip (60 mm × 24 mm × 0.17 mm), and chromosome slide was immediately put horizontally into warm water vapors (65–70 °C). After air drying slide was rinsed in phosphate buffer (1xPBS, pH = 7.2) for 1 min at RT and immediately transferred in 0.1 % Giemsa solution for 3–4 min at RT. After staining, the slide was rinsed in distilled water and slightly dried. The chromosomal material should remain wet and soft for easy and careful its collection without breaking into fragments with extended siliconized glass needle. This procedure of chromosome preparation for microdissection reduced DNA degradation and allowed the quantitative collection of chromosome material.

Microdissection was carried out on an AxioVert10 inverted microscope (ZEISS, Germany) equipped with two micromanipulators, one of which controlled an extended glass needle. At the same time, the other served to fix the Pasteur pipette with an extended tip during the transfer of dissected material. For more efficient microdissection, a special rotating sliding stage was installed on AxioVert10 inverted microscope.

Material of all chromosomes from the selected metaphase plate was collected and transferred under microscopic control into reaction mixture solution in the extended siliconized tips of the Pasteur pipettes (the diameter of the tip was about 40 μm). Then it was transferred into the separate PCR tubes contained 10 μl of the reaction mixture (Zadesenets et al., 2016). To ensure that the material was transferred completely, we broke off, the extended tip of the Pasteur pipette in the PCR tube. Further, the preparation of DNA for amplification (proteinase K treatment, DNA fragmentation, DNA library preparation) and DNA amplification itself were carried out according to the standard protocol (Zadesenets et al., 2016, 2017a, b, 2020). The resulting PCR products were labeled, and two-color CISS-hybridization was performed for testing the quality of the obtained WCPs and to determine WCPs generated from homologous chromosomes.

**Fig. 2.**
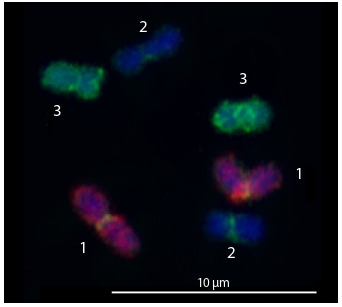
CISS-hybridization with the WCPs derived from chromosome 1 (red signal) and chromosome 3 (green signal) of M. cliftonensis on the metaphase chromosomes of M. cliftonensis. Chromosome numbers are indicated.

The CISS-hybridization with obtained WCPs painted entirely one pair of chromosomes and gave a signal in the pericentromeric regions of other chromosomes (Fig. 2). The last could be provided by insufficient suppression of repetitive DNA hybridization. The DNA libraries generated from homologous chromosomes were pooled together. After CISShybridization, the WCPs based on the combined DNA libraries gave more intense and more specific signal on the original chromosome. They also provided more intense FISH signal at the pericentromeric regions of all chromosomes. At the same time, the painting intensity at the euchromatic regions of other chromosomes did not increase. Two-color CISS-hybridization with obtained WCPs did not reveal chromosome translocation in the M. cliftonensis karyotype. These results confirmed our previous suggestion that the M. cliftonensis karyotype is highly stable.

**Fig. 3.**
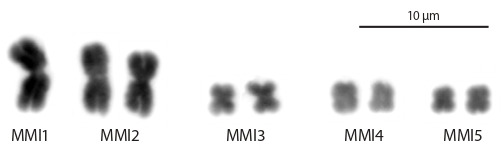
The karyotype M. mirumnovem (2n = 9) consists of three large chromosomes and three pairs of small metacentric chromosomes showing similar size and morphology.

**Generation and testing of WCPs derived from metaphase chromosomes of M. mirumnovem.** Since we uncovered high karyotype instability in the laboratory culture of M. mirumnovem (Zadesenets et al., 2020), the worms with the 2n = 9 karyotype were chosen for the WCP generation. At the beginning of the cultivation of M. mirumnovem worms under the laboratory conditions, the most common karyotype revealed among the specimens was 2n = 9 (Fig. 3). The same protocol of metaphase chromosome microdissection and sequence-independent DNA amplification described for M. cliftonensis was applied for generation of the WCPs from the M. mirumnovem chromosomes. The material of all nine chromosomes was isolated separately from one metaphase plate, and the WCPs were obtained by DNA amplification of the collected material. The following CISS-hybridization was performed on metaphase chromosomes of M. mirumnovem, and pairs of WCPs derived from homologous chromosomes were determined. Microdissected DNA libraries obtained from homologous chromosomes were pooled together and were further used for the production of the WCPs. As a result, we received the set of WCPs that includes four WCPs derived from the MMI2-MMI5 chromosome pairs and one WCP derived from one copy of unpaired chromosome MMI1.

**Fig. 4.**
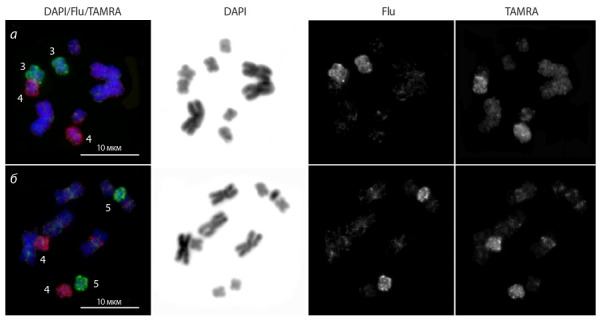
CISS-hybridization with the WCPs on metaphase chromosomes of M. mirumnovem. а – the whole chromosome paints derived from small chromosomes MMI3 (green signal) and MMI4 (red signal); b – the whole chromosome paints derived from small chromosomes MMI4 (red signal) and MMI5 (green signal). Chromosome numbers are indicated.

CISS-hybridization of WCPs obtained from small chromosomes MMI3–MMI5 gave intensive and specific fluorescent signals on the original chromosome, less intensive signal at the pericentromeric regions of the remaining chromosomes, and weak non-specific signals at the euchromatic regions of other small metacentrics. However, the painting pattern of large chromosomes appeared to be more complicated for interpretation. The WCPs obtained from chromosomes MMI3– MMI5 painted specifically but less intensively and unevenly different regions of the MMI2 chromosome, and they painted even less intensively and less evenly the MMI1 chromosome (Fig. 4).

CISS-hybridization with the WCPs derived from the large chromosome MMI1 and two copies of the MMI2 painted intensively the original chromosomes. However, the pattern of chromosome painting was uneven. On low condensed chromosomes, areas of intense fluorescence alternated with less intensely painted regions. We should note that CISS-hybridization with the WCPs derived from large metacentrics MMI1 and MMI2 gave weak specific fluorescent signals on small metacentric chromosomes MMI3–MMI5. We believe that the obtained painting patterns of the M. mirumnovem chromosomes indicate to the WGD event in the evolutionary scenario of this species. However, it has been accompanied by intensive genome and karyotype reorganization, possibly leading to the rediploidization of the modern M. mirumnovem genome. A similar scenario of genome and karyotype evolution was previously described for the other Macrostomum species, M. lignano and M. janickei, belonging to another phylogenetic lineage (Schärer et al., 2020; Zadesenets et al., 2020).

## Conclusion

The proposed and tested approach for the preparation of DNA probes from individual whole chromosomes allowed us to obtain the WCPs for chromosomes of M. cliftonensis and M. mirumnovem. The generated WCPs efficiently identified the material of their original chromosomes in both species. Moreover, in the M. mirumnovem chromosomes, the WCPs revealed the paralogous regions, resulting from the recent WGD followed by subsequent reorganization of ancestral chromosomes.

## Conflict of interest

The authors declare no conflict of interest.
